# Factors Associated with the Patient/Client Use of Report Cards, Physician Rating Websites, Social Media, and Google for Hospital and Physician Selection: A Nationwide Survey

**DOI:** 10.3390/healthcare10101931

**Published:** 2022-10-01

**Authors:** Tsung-Tai Chen, Chyi-In Wu, Ming-Hsin Phoebe Chiu, Jia-Lien Hsu, Mao-Hung Liao, Ya-Seng Arthur Hsueh, Wei-Chih Su

**Affiliations:** 1 Department of Public Health, College of Medicine, Fu Jen Catholic University, New Taipei 242, Taiwan; 2 Institute of Sociology, Academia Sinica, Taipei 115, Taiwan; 3 Graduate Institute of Library and Information Studies, National Taiwan Normal University, Taipei 106, Taiwan; 4 Department of Computer Science and Information Engineering, College of Science and Engineering, Fu Jen Catholic University, New Taipei 242, Taiwan; 5 Yonghe Cardinal Tien Hospital, New Taipei 234, Taiwan; 6 Centre for Health Policy, Melbourne School of Population and Global Health, The University of Melbourne, Melbourne, VIC 3010, Australia; 7 Department of Gastroenterology, Buddhist Tzu Chi Medical Foundation, Taipei Tzu-Chi Hospital, New Taipei 231, Taiwan

**Keywords:** physician rating websites (PRWs), social media, Google, nationwide survey, influencing factors

## Abstract

Objective: To explore the factors associated with the different uses of report cards, physician rating websites, social media, and Google, including awareness, physician finding, and decision-making based on reviews from the patient/client perspective. Methods: We used computer-assisted telephone interviews to conduct a nationwide representative survey in Taiwan. Results: The urbanization level of the area, income, and long-term health conditions were not associated with the three kinds of usage of the websites studied. Seeking health information was an important factor in the three kinds of website use. The employment industry was associated with awareness, and education level was associated with physician seeking and actions based on reviews. Conclusions: Different factors influenced the three kinds of usage: awareness, actual use (i.e., finding an appropriate physician), and decision-making based on reviews. Seeking health information is of primary importance regardless of how the websites are used. Practical implications: Policy-makers should focus on educating individuals working outside the health care sector to increase awareness of these websites and to assist individuals with low levels of education in increasing their use of these websites.

## 1. Introduction

Public report cards traditionally represent public disclosure of subjective quality information on performance measures (e.g., infection rates) for facilities, which leads to improvements in quality of care from the facility side and steers patients toward high-quality providers (foot voting) from the patient side [1]. In the era of Medicine 2.0, over the past 10 years, patients have gradually become used to evaluating hospitals and physicians, sharing their medical experience narratives, and communicating with other patients on health-related websites [2]. Generally, these medical evaluations based on personal experience exist on physician rating websites (PRWs), social media (e.g., Facebook), and Google platforms. Research has shown that patients prefer not to use official quality report cards of medical institutions, which mainly use objective measures, including complication rates or surgical volume [3]. Instead, they prefer to use commercial or noncommercial PRWs or social media as a reference when choosing physicians [4,5,6,7,8,9,10,11,12,13,14,15,16,17,18,19]. Patients can easily observe and judge physicians’ interpersonal quality via PRWs, and these websites are usually user-friendly and provide ample information in an easily understandable format [20,21].

Regarding the use of these websites, most studies have explored the influencing factors related to only one or two of the four kinds of usage: awareness [14,22], decision-making, [14,23,24,25], response to the results (i.e., visiting or not visiting a physician) [14], and leaving a review after visiting a physician (feedback) [25,26,27,28]. Many studies related to these four uses of websites have employed paper surveys [22], online surveys [14,24,29], and interviews [23]. Most of these studies were conducted with non-Asian populations, and few studies have used representative nationwide samples, limiting the external generalizability of their results and leading to failure to design appropriate and effective policies that target key factors. Furthermore, an increasing number of people use reviews posted on Google when selecting a physician [30], and research has indicated that Facebook reviews are somewhat reliable [31]. A Swiss study found that more people left feedback about physicians on Google than on several Swiss PRWs [30]. Beyond PRWs, the Pew Research Center found that 68% of American adults had ever used Facebook [32], and social media traffic is much greater than that for official quality report cards [33]. Clearly, Google and social media have become important and preferred sources of medical experience narratives among patients. However, few studies have explored the associated factors that influence the four kinds of use for Google and social media.

In this study, we conducted a nationwide survey to explore factors associated with the use of these websites, including public report cards, PRWs, social media, and Google. The four kinds of uses we examined were awareness of the websites, actual use (i.e., finding an appropriate physician), response to the results, and leaving a review.

## 2. Methods

### 2.1. Computer-Assisted Telephone Interview (CATI) Methodology and Sample Selection Criteria

This nationwide survey used a cross-sectional CATI methodology implemented by the Center for Survey Research, Academia Sinica [34] to conduct a survey of 1250 citizens across Taiwan from 20 July to 25 July 2020.

To minimize the chance of sampling invalid numbers and thus reduce the time and resource investment, the survey was conducted by sampling mobile phone numbers nationwide through random digit dialing (RDD) using stratified two-stage probability proportional to size (PPS) systematic sampling. In the first stage, four strata were created based on the use volume of the first 6 digits of the mobile phone number. The determination of the sample size of phone numbers for each stratum was proportional to the average use rate of four strata. Then, the mobile phone numbers were ranked in ascending order according to their use volume in each stratum, and the use volume of every mobile phone number was accumulated. In the second stage, mobile phone numbers were chosen based on systematic sampling from each of the four strata. In this stage, the six-digit mobile phone numbers were randomly sampled using the last three digits [35].

### 2.2. Recruitment

The inclusion criteria for the participants were (1) any use of the internet within the past year; (2) having a mobile phone; and (3) being 20 to 60 years of age.

### 2.3. Questionnaire and Outcomes

The validity of the questionnaire was checked by five experts, and a pilot study of 100 persons was conducted via the CATI method. The questionnaire consisted of four parts focused on awareness, usage, decision-making, and review-writing with respect to PRWs, social media (Facebook and local forums (i.e., PTT or Mobile01)) and Google. The relevant items in the questionnaires were the following. (1) Which report cards or PRWs are you aware of? (multiple choices) (2) Actual use of report cards, PRWs, or social media/Google to search for quality information; (3) Decision making based on report cards or reviews on PRWs, social media, or Google search engine after actual use of report cards, PRWs, or social media (or the Google search engine); (4) Posting a review after visiting a physician (feedback). Because the four kinds of uses are correlated, a participant who reported awareness of a specific website could answer the question related to the use of this website to find an appropriate physician/facility. Additionally, he or she could answer the question related to decision-making based on the results of this website.

### 2.4. Statistical Analysis

We used a generalized linear mixed model (GLMM) with Laplace estimation to derive the models by using the factors below. Observations with missing variables were overlooked in these models (28 observations). The fixed effects were tested with a Wald *t*-test. The four outcomes related to these websites, awareness, usage, responses to reviews, and review writing, were fitted to the Bernoulli distribution (binary data). Individual-level variables were added as fixed effects using backward elimination, and nonsignificant variables were sequentially removed until only significant (*p* < 0.05) variables remained. To confirm that the final GLMM model was appropriate, we checked whether the residuals were approximately normally distributed. In addition, we used the weighting data for a sensitivity analysis. The weighting was based on the population between the ages of 20 and 60 in Taiwan, which totaled 14,073,983 [36]. The details of the weighting method are presented in Supplementary S1. All estimations were derived using SAS version 9.4 (Statistical Analysis Systems, Inc., Cary, NC, USA).

### 2.5. Controlled Factors

We controlled for two levels of factors: individual and regional socioeconomic status. The individual factors were categorized as demographic factors, use of the internet, and clinical factors. The details of the controlled factors are presented in Supplementary S2.

## 3. Results

In Table 1, the final sample included 1250 cases. The sampling error with a 95% confidence interval was ±2.77%. The response rate (RR5), as defined by the American Association for Public Opinion Research (AAPOR), was 81.06. Participant characteristics are described in Supplementary S3.

### 3.1. Factors Related to Awareness of Official Report Cards or PRWs

In Table 2, regarding awareness of official report cards or other PRWs, an area-level factor (high degree of urbanization) was not significantly associated with high awareness of these websites. Individual factors, including younger age, were significantly associated with low awareness of official report cards (20~29: OR = 0.55, *p* = 0.02; 30~39: OR = 0.55, *p* = 0.02). Regarding employment industry, for official report cards, the manufacturing (OR = 0.22, *p* < 0.001), construction (OR = 0.41, *p* = 0.04), wholesale and retail (OR = 0.28, *p* = 0.003), accommodation and catering (OR = 0.33, *p* = 0.01), and other (OR = 0.30, *p* < 0.001) industries as well as unemployment (OR = 0.41, *p* = 0.01) were significantly more strongly associated with low awareness than the health care and social work industry; the results were similar to those for private PRWs. Regarding education, for official report cards, participants with a general university degree were associated with significantly lower awareness than those with a vocational high school diploma or below (OR = 0.59, *p* = 0.04), and participants with no long-term conditions were significantly less aware of official report cards (OR = 0.58, *p* = 0.01) and private PRWs (OR = 0.60, *p* = 0.008). Health information seeking online was significantly associated with high awareness of official public report cards (OR = 1.92, *p* = 0.01) and PRWs (OR = 1.88, *p* = 0.009).

### 3.2. Factors Related to the Use of Report Cards, PRWs, Social Media or Google

As shown in Table 3, the area-level factor degree of urbanization was not significantly associated with the use of official report cards, private PRWs, or social media (or Google). Younger participants, including those aged 20~29 (OR = 2.39, *p* < 0.001), 30~39 (OR = 2.49, *p* < 0.001), and 40~49 (OR = 1.63, *p* = 0.009), reported significantly higher use of social media or Google than those aged 50~60. Male sex was significantly associated with low use of social media/Google (OR = 0.72, *p* = 0.01). Participants with a high level of education were significantly more likely to report high use of PRWs or social media/Google. Participants with a junior college degree (OR = 2.23, *p* = 0.01), a general university degree (OR = 2.58, *p* < 0.001), or a graduate degree (OR = 3.24, *p* < 0.001) reported significantly higher use of PRWs than participants with a vocational high school diploma or below. Similar results were found for the use of social media or Google. Participants with a junior college degree (OR = 1.59, *p* = 0.03), a technical or military university degree (OR = 1.61, *p* = 0.02), a general university degree (OR = 1.59, *p* = 0.01), or a graduate degree (OR = 2.60, *p* < 0.001) reported significantly higher use of these websites. Being married was associated with significantly higher use of social media or Google than being unmarried (OR = 1.38, *p* = 0.03). Daily internet use was significantly associated with high use of social media or Google (OR = 5.43, *p* < 0.001), and health information seeking was also significantly associated with three websites: official report cards (OR = 4.97, *p* = 0.03), PRWs (OR = 27.51, *p* = 0.001), and social media (or Google) (OR = 4.40, *p* < 0.001).

### 3.3. Factors Related to Action Based on Feedback from Report Cards, PRWs, Social Media Networks, Google, and Reviews from All except Report Cards

None of the variables were associated with the response to search results for the official report cards and PRWs (data not shown). As shown in Table 3, the area-level factor degree of urbanization was not significantly associated with the use of these websites. Younger respondents, including those aged 20~29 (OR = 2.18, *p* < 0.001) and 30~39 (OR = 2.31, *p* < 0.001), had a significantly stronger response to the results of a social media or Google search than those aged 50~60. Male sex was significantly associated with a weak response to the results of a social media or Google search (OR = 0.67, *p* = 0.005). A high level of education was significantly associated with a strong response to the results of a social media or Google search. Participants with a junior college degree (OR = 2.06, *p* = 0.006), technical or military university degree (OR = 1.90, *p* = 0.007), general university degree (OR = 2.18, *p* < 0.001), or graduate degree (OR = 3.83, *p* < 0.001) had a significantly stronger response to the results of a social media or Google search than those with a vocational high school diploma or below. Daily internet use was significantly associated with a higher likelihood of acting based on the results of a social media or Google search (OR = 5.41, *p* = 0.02), as was health information seeking (OR = 4.14, *p* < 0.001). Finally, we could not derive any factors related to leaving reviews on PRWs, social media networks or Google (data not shown).

All of the above regression results held after the weighting factor was applied. The results are shown in Tables S1 and S2 of Supplementary S4.

## 4. Discussion and Conclusions

### 4.1. Discussion

This is the first nationwide study with rigorous random sampling conducted in Taiwan to investigate the factors associated with the awareness and use of technical measures in official report cards, reviews on PRWs, and social media (or the Google search engine) to find a physician/facility and responses to reviews on these websites. The participants surveyed are similar to the population demographics of Taiwan in terms of age and gender after applying a weighting factor (data not shown), thus demonstrating the validity of this study.

Although we could not measure the factors that influence reviewing, we found that different factors influence the other three kinds of usage before the reviewing stage: awareness, use in decision-making, and response to the results. In general, the environment (urbanization level of the area of residence), income and long-term health conditions are not important factors for the three kinds of uses for these websites. Seeking health information is an important factor in all three kinds of usage. The employment industry is associated with awareness of report cards and PRWs; however, education level is associated with the use of report cards, PRWs, Google and Facebook and action based on the information on these websites.

### 4.2. Explanations of Factors Affecting Awareness of Official Report Cards and PRWs

Previous studies have shown that female sex, [14] being widowed, [14] high health care utilization or poor health status, [14] high internet use, [14] and health information seeking [14,22] are associated with high awareness of report cards and PRWs. Based on our results, which were derived from nationwide sampling data and verified by a rigorous method (the GLMM and weighting factor), we found that health information seeking is a critical factor and that employment in the health care industry is associated with higher awareness of report cards and PRWs than employment in other industries, especially the manufacturing, wholesale and retail industries. In addition, we found that participants with long-term conditions (chronic diseases) had significantly higher awareness of official report cards or private PRWs; however, this factor was not significantly associated with the subsequent use of any website. Potential reasons are as follows: (1) patients with long-term conditions may use PRWs mainly to obtain information about treatment and drugs [27] and not to find appropriate physicians, and (2) patients with high-risk but nonchronic diseases may be more willing to use these websites to select physicians, as shown in previous research [37].

### 4.3. Explanation of Factors for Usage and Decision Making Based on the Results of PRWs, Social Media Networks or Google

Previous studies have shown that a higher education level [25], a low level of income [23], high health care utilization or poor health status [14,25], and high internet use [14] were associated with a high use of PRWs. Another study related to the use of mobile physician-rating apps showed that younger age, daily internet use for health-related information, and the frequency of using apps for health-related information in the past were associated with the adoption of mobile physician-rating apps [24]. Regarding responses to the results of PRWs, previous studies have shown that high health care utilization and high internet use were associated with high responsiveness to PRWs [14].

We found that inferior neighborhood conditions, income level and long-term health conditions were not associated with PRW, social media, or Google use or with acting based on the information on these websites. The vital factor for using these websites and acting based on the results was health information seeking. Previous research has shown that increasing numbers of patients seek health information online, [38] and this behavior can make them feel empowered and encouraged to participate in decision-making [39]. Conflicts, dissatisfaction and arguments may occur between these patients and their physicians due to the receipt of health information that is not aligned with physicians’ suggestions, [40] and patients may find recommendations on the internet [41]. In addition, we found that the second most important factor, high education level, was associated with PRW, social media, and Google use and with acting based on the information on these websites. High levels of education are associated with high levels of health education; hence, citizens with high health literacy are more likely to better understand and operate relevant services and have the ability to assess the credibility of retrieved information for making decisions [42,43].

This study has several limitations. First, we could not discuss some factors related to the use of the studied websites, such as cognitive variables, physician trust, website quality, and special contexts [37,44,45,46,47,48]. However, we used important demographic, socioeconomic, internet-related, behavioral, and clinical factors to comprehensively interpret the three kinds of uses of official report cards, PRWs, Google searches and Facebook, including awareness, finding appropriate physicians/facilities, and action taken based on the results of these websites. Future studies should consider all factors that may be associated with the uses of these websites. Second, in this study, we only reported the results of influencing factors associated with the three kinds of uses of these websites but not factors associated with review-leaving behavior. A possible reason is that only 57 respondents (5%) left reviews on any website in this study (data not shown). This number of cases is not sufficient because of the limited research budget for nationwide surveys. In the future, the sample size should be enlarged so that review-leaving behavior can be further investigated. In addition, in the future, we can conduct data analysis from PRWs, social media or Google reviews by using data mining or text mining in order to understand factors associated with review-leaving behavior. Third, we do not have any recent report that demonstrates a neighborhood or regional breakdown that is similar between the ages of 20 to 60 of the population. However, we confirm that the overall population between the aforementioned age range in Taiwan is sampled with equal probability in this study, because of the PPS systematic sampling method if a person has at least one mobile phone.

### 4.4. Conclusions

Different factors influenced the three kinds of usage: awareness, actual use (i.e., finding an appropriate physician), and decision-making based on reviews. Seeking health information is of primary importance regardless of how the websites are used. However, employment in different industries is key for awareness, and the level of education is vital for the actual use of and responsiveness to the reviews on these websites.

### 4.5. Practical Implications

If policy-makers want to help citizens use report cards, PRWs, Google, and social media efficiently, including awareness, finding appropriate physicians/facilities, and actions based on these results, they should first focus on educating individuals working outside of the health care sector to increase awareness of report cards and PRWs and then assisting individuals with low levels of education in increasing their use of these websites.

## Figures and Tables

**Table 1:** Characteristics of the study participants.

	No (%)
**Total *n***	1250 (100)
**Age (years)**	
20~29	340 (27)
30~39	336 (27)
40~49	314 (25)
50~60	256 (21)
Missing	4 (0.3)
**Male**	672 (54)
**Employment industry** ^a^	
Manufacturing	235 (19)
Construction	83 (7)
Wholesale and retail	108 (9)
Accommodation and catering	86 (7)
Health care and social work	78 (6)
Other	444 (36)
Unemployment	209 (17)
Missing	7 (1)
**Education**	
Vocational high school or below ^b^	370 (30)
Junior college ^c^	155 (12)
Technical or military university ^d^	235 (19)
General university	300 (24)
Graduate school or doctorate	190 (15)
**Marital status**	
Never	520 (42)
Married	631 (50)
Divorced	65 (5)
Widowed	24 (2)
Separated	8 (2)
Missing	2 (0.2)
**Health information seeking**	988 (79)
**Internet use per day last week**	1190 (95)
**Long-term health conditions**	232 (19)
**Level of urbanization**	
High-level	360 (29)
Median-level	396 (32)
Emerging	265 (21)
Common	148 (12)
Aging	12 (1)
Agricultural	22 (2)
Remote areas	31 (3)
Missing	16 (1)

Note: Industry ^a^: The standard industry contains 19 categories. We list only the top 5 industries and place the other 15 industries in the ‘other’ variable; Vocational high school or less ^b^: includes citizens who cannot read, are self-taught, or attended elementary school, junior high school, a general education senior high school, or a vocational senior high school; Junior college ^c^: includes 5-year, 3-year, 2-year, or open junior colleges or open universities; Technical or military university ^d^: includes institutes of technology, universities of technology, military schools, or national defense universities.

**Table 2:** Factors related to awareness of official report cards or private PRWs (*n* = 1222).

	Official Report Card	Private PRWs
	ORs (95% CI)	ORs (95% CI)
**Area-level**		
Degree of urbanization	0.96 (0.84, 1.09)	1.04 (0.92, 1.18)
**Individual-level**		
Age (Ref: 40~49 years)		
*20~29*	0.55 (0.33, 0.92) *	
*30~39*	0.55 (0.34, 0.90) *	
Employment industry (Ref: Health care and social work)		
*Manufacturing*	0.22 (0.11, 0.46) ***	0.18 (0.09, 0.34) ***
*Construction*	0.41 (0.17, 0.95) *	0.30 (0.14, 0.66) **
*Wholesale and retail*	0.28 (0.12, 0.66) **	0.27 (0.13, 0.55) ***
*Accommodations and catering*	0.33 (0.13, 0.79) *	0.27 (0.12, 0.59) **
*Other*	0.30 (0.16, 0.57) ***	0.25 (0.14, 0.42) ***
*Unemployment*	0.41 (0.21, 0.81) *	0.30 (0.17, 0.56) ***
Education (Ref: Vocational high school or below ^a^)		
*General university*	0.59 (0.35, 0.98) *	
No long-term condition	0.58 (0.38, 0.88) *	0.60 (0.41, 0.87) **
Health information seeking	1.92 (1.14, 3.25) *	1.88 (1.16, 3.04) **

Notes: Ref = reference; CI = confidence interval; Vocational high school ^a^: include citizens who cannot read, are self-educated, or attended elementary school, junior high school, a general education senior high school, or a vocational senior high school. * *p* < 0.05; ** *p* < 0.01; *** *p* < 0.001.

**Table 3:** Factors related to the use of official report cards, private PRWs, social media or Google and factors related to action based on social media or Google (*n* = 1222).

			Social Media or Google ^a^	
	Use of Report Card	Use of PRWs	Use	Action/Response
	ORs (95% CI)	ORs (95% CI)	ORs (95% CI)	ORs (95% CI)
**Area-level**				
Degree of urbanization	1.01 (0.79, 1.29)	1.02 (0.87, 1.19)	1.07 (0.97, 1.18)	1.09 (0.97, 1.22)
**Individual-level**				
Age (Ref: 50~60 years)				
*20~29 years*			2.39 (1.51, 3.76) ***	2.18 (1.51, 3.15) ***
*30~39 years*			2.49 (1.69, 3.69) ***	2.31 (1.65, 3.24) ***
*40~49 years*			1.63 (1.13, 2.37) **	
Male			0.72 (0.56, 0.93) *	0.67 (0.51, 0.91) **
Education (Ref: Vocational high school or below ^b^)				
*Junior college* ^c^		2.23 (1.19, 4.17) *	1.59 (1.04, 2.43) *	2.06 (1.23, 3.45) **
*Technical military university* ^d^			1.61 (1.08, 2.39) *	1.90 (1.19, 3.01) **
*General university*		2.58 (1.55, 4.29) ***	1.59 (1.10, 2.31) *	2.18 (1.41, 3.37) ***
*Graduate school or doctorate*		3.24 (1.89, 5.56) ***	2.60 (1.73, 3.92) ***	3.83 (2.42, 6.05) ***
Marriage (Ref: unmarried)				
*Married*			1.38 (1.03, 1.85) *	
Internet use per day			5.43(2.06, 14.35) ***	5.41 (1.26, 23.17) *
Health information seeking	4.97 (1.19, 20.83) *	27.51(3.80, 199.16) **	4.40 (3.03, 6.37) ***	4.14 (2.50, 6.88) ***

Note: None of the variables were associated with actions based on the results of official report cards and PRWs due to the small sample size (*n* = 51); Social media or Google ^a^: use of any social media platform or the Google search engines; Vocational high school or below ^b^: includes citizens who cannot read, are self-taught, or attended elementary school, junior high school, a general education senior high school, or a vocational senior high school; Junior college ^c^: includes 5-year, 3-year, 2-year, or open junior colleges or open universities; technology or military university ^d^: includes institutes of technology, universities of technology, military schools, or national defense universities. * *p* < 0.05; ** *p* < 0.01; *** *p* < 0.001.

## Data Availability

The data presented in this study are available on request from the corresponding author. The data are not publicly available due to data restriction policies.

## Supplementary Materials

The following are available online at https://www.mdpi.com/article/10.3390/healthcare10101931/s1. Supplementary S1: The details of the weighting method; Supplementary S2: The details of the controlled factors; Supplementary S3: Description of participant characteristics; Supplementary S4: Table S1. Awareness of official report cards or private physician rating websites; Table S2. Use of official report cards, private physician rating websites, social media networks a or Google and action based on social media a or Google.

## Abbreviations

PRW	physician rating website
NHI	national health insurance
NHIA	National Health Insurance Administration
CATI	computer-assisted telephone interview
RDD	random digit dialing
PPS	probability proportional to size
GLMM	generalized linear mixed model
ISIC	International Standard Industrial Classification
TCS	Taiwan Communication Survey
NHRI	National Health Research Institute
AAPOR	American Association for Public Opinion Research
