# Supplementary material for: Factors Associated with the Patient/Client Use of Report Cards, Physician Rating Websites, Social Media, and Google for Hospital and Physician Selection: A Nationwide Survey
Source: Healthcare (Basel). 2022 Oct 1;10(10):1931. doi: 10.3390/healthcare10101931 (PMC9602070; doi:10.3390/healthcare10101931)
Supplement: Supplementary file 1 [file healthcare-10-01931-s001.zip › healthcare-1895569-supplementary.pdf]

Supplementary S1

The weighting method

First, to ensure an equal probability of selection, we applied unequal probabilities of selection (sampling weights) to adjust the chance of being selected as samples in our study because of multiple mobile phone numbers for one person (f_c). We considered a person who had multiple mobile phone numbers as a weighting factor and inversed the weighting factor as a weight for unequal probabilities of selection (w_{sel}^{0}), which equaled $w_{sel}^{0} = \frac{1}{f_c}$. The final weighting for unequal probabilities of selection that was adjusted to the proportion of the sample size in our study (*n*) to analyze the expected probability of being selected as a sample (N_v, sum of w_{sel}^{0} for every sample) was calculated as follows:

$$w_{sel} = w_{sel}^{0} \times \frac{n}{Nv}$$

Second, to ensure that our sample met the distributions in the total population in Taiwan in terms of age and sex variables, we applied a multiple iterative weighting method to weight the samples until the sample distributions of these two variables met the distributions in the total population with significant results in the chi-squared test. The multiple iterative weighting (w_{fin}) was calculated as follows:

$$w_{fin} = w_{sel} \times \frac{N_i}{N} \times \frac{n}{n_i}$$

N =total population size, n=sample size in this study,
N_i=sex and age distributions in the total population,
n_i= sex and age distributions in our sample

Supplementary S2

We controlled for two levels of factors: individual and regional socioeconomic status. The individual factors were categorized as demographic factors, use of the internet, and clinical factors.

The demographic factors included age, sex, employment industry, education (including current students and dropouts), and marital status. Age was divided into 4 groups: 20-29 years, 30-39 years, 40-49 years, and 50-60 years. The employment industry comprised 19 categories based on the International Standard Industrial Classification (ISIC) of All Economic Activities of the Directorate-General of Budget, Accounting and Statistics, Executive Yuan. The education classification comprised 21 categories based on the Taiwan Communication Survey (TCS). Marital status was divided into married, never married, divorced, widowed, or separated. The use of the internet included ever having searched for health information online (yes or no) and internet use per day in the previous week (including LINE or Facebook usage) (yes or no). Clinical factors included having a long-term condition that had been diagnosed

by any physician, such as diabetes, heart disease, hypertension, asthma, or depression (yes or no).

Urbanization of the area of residence was used as a proxy for regional socioeconomic status. The communities in Taiwan were stratified into 7 urbanization categories according to the standard published by the Taiwanese National Health Research Institute (NHRI), which used cluster analysis to divide them into urbanization groups from the highest level of urbanization to the lowest: high-level, median-level, emerging, common, aging, agricultural, and remote areas. If the town was located in an area with a high level or urbanization, we coded it as 7. A higher score indicated that the town had a higher degree of urbanization.

Supplementary S3

Table 1 in the main text shows that approximately 27% (340/1250) of participants were 20-29 years old, 27% (336/1250) were 30-39 years old, 25% (314/1250) were 40-49 years old, and 21% (256/1250) were 50-60 years old. The top 5 industries in which participants were employed were manufacturing (19%, 235/1250), construction (7%, 83/1250), wholesale and retail (9%, 108/1250), accommodation and catering (7%, 86/1250), and health care and social work (6%, 78/1250); these industries accounted for approximately 70% of participants who were employed. These

industries were used in the subsequent regression analysis, and other industries were categorized as other. Regarding education, there were 5 groups: vocational high school or below, junior college, technical or military university, general university, and graduate school. Of the respondents, 40% had less than a university education, including 30% (370/1250) with a vocational high school diploma or below and 12% (155/1250) with a junior college degree. Fifty percent of respondents were married (631/1250), and 79% (988/1250) had ever searched for health information. A total of 95% (1190/1250) of respondents used the internet every day in the previous week, 19% (232/1250) had long-term health conditions, and 29% (360/1250) lived in a town with a moderate level of urbanization or above.

Supplementary S4

Table S1 Awareness of official report cards or private physician rating websites (*n*=1,212)

	All<br>ORs (95% CI)	Official report card<br>ORs (95% CI)	Private PRWs<br>ORs (95% CI)
**Area level**			
Degree of urbanization	1.13 (1.03, 1.24)*	0.97(0.85, 1.11)	1.07 (0.94, 1.21)
**Individual level**			
Male	0.68 (0.52, 0.89)**		
Age (Ref: 40~49 years)			
20~29		0.48 (0.27, 0.84)*	
30~39		0.51 (0.31, 0.83)**	
Employment industry (Ref: Health care and social workers)			
Manufacturing	0.37 (0.21, 0.68)**	0.22 (0.11,0.46)***	0.18 (0.09, 0.34)***
Construction	0.41 (0.20, 0.84)*	0.41 (0.17, 0.95)*	0.30 (0.14, 0.65)**
Wholesale and retail	0.35 (0.18, 0.67)**	0.26 (0.11, 0.62)**	0.25 (0.12, 0.51)***
Accommodation and catering	0.36 (0.18, 0.73)**	0.33 (0.14, 0.81)*	0.25 (0.11, 0.56)***
Others	0.36 (0.20, 0.63)***	0.31 (0.17, 0.58)***	0.24 (0.14, 0.42)***
Unemployment	0.29 (0.16, 0.52)***	0.44 (0.22, 0.88)*	0.29 (0.15, 0.53)***
Education level (Ref: Vocational high school or below [a])			
General university		0.55 (0.33, 0.92)*	
Graduate school or doctorate	1.69 (1.20, 2.36)**		
No long-term condition		0.60 (0.39, 0.92)*	0.61 (0.41, 0.89)*
Internet use per day	0.49 (0.26, 0.93)*		0.47 (0.23, 0.98)*
Health information seeking	4.73 (3.25, 6.88)***	1.86 (1.10, 3.14)*	2.00 (1.22, 3.28)**

Notes: Ref = reference; CI = confidence interval; *P < 0.05; **P < 0.01; ***P < 0.001.Vocational high school [a]: includes citizens who cannot read, are self-taught, or attended elementary school, junior high school, a general education senior high school, or a vocational senior high school

Table S2 Use of official report cards, private physician rating websites, social media networks [a] or Google and action based on social media [a] or Google (*n*=1,222)

			Usage	Action/response
	Use of report card	Use of PRWs	Social media or Google [a]	Social media or Google [a]
	ORs (95% CI)	ORs (95% CI)	ORs (95% CI)	ORs (95% CI)
**Area level**				
Degree of urbanization	1.01 (0.79, 1.29)	1.02 (0.87, 1.19)	1.07 (0.97, 1.17)	1.09 (0.97, 1.22)
**Patient level**				
Age (Ref: 50~60 years)				
20~29 years			2.15 (1.29, 3.57)**	2.14 (1.42, 3.23)***
30~39 years			2.30 (1.50, 3.53)***	2.28 (1.59, 3.26)***
40~49 years			1.55 (1.05, 2.29)*	
Male			0.69 (0.53, 0.90)**	0.67 (0.50, 0.89)**
Education level				
(Ref: Vocational high school or below [b])				
Junior college [c]		2.23 (1.19, 4.17)*	1.60 (1.05, 2.44)*	2.06 (1.23, 3.44)**
Technical or military university [d]			1.59 (1.07, 2.37)*	1.89 (1.19, 3.00)**
General university		2.58 (1.55, 4.29)***	1.58 (1.09, 2.30)*	2.17 (1.40, 3.36)***
Graduate school or doctorate		3.24 (1.89, 5.56)***	2.57 (1.70, 3.88)***	3.81 (2.40, 6.03)***
Marriage (Ref: unmarried)				
Married			1.40 (1.04, 1.87)*	
Internet use per week			5.44 (2.06, 14.39)***	5.39 (1.26, 23.02)*
Health information seeking	4.97 (1.19, 20.83)*	27.51 (3.80, 199.16)**	4.38 (3.02, 6.35)***	4.14 (2.49, 6.84)***

Note: None of the variables were associated with action based on the results of official report cards and PRWs due to the small sample size (n=51); Social media or Google [a]: use of any social media platform or the Google search engine; Vocational high school or below [b]: includes citizens who cannot read, are self-taught, or attended elementary school, junior high school, a general education senior high school, or a vocational senior high school; Junior college [c]: includes 5-year, 3-year, 2-year, open junior colleges or open universities; Technical or military university [d]: includes institutes of technology, universities of technology, military schools, or national defense universities. *$P < 0.05$; **$P < 0.01$; ***$P < 0.001$
